# NTRK rearranged sarcoma of the bone. Role for larotrectinib in the neoadjuvant setting of an ultra-rare tumor: a case report

**DOI:** 10.3389/fonc.2023.1252359

**Published:** 2023-10-09

**Authors:** Emanuela Palmerini, Giorgio Frega, Marco Gambarotti, Tommaso Frisoni, Marilena Cesari, Alberto Bazzocchi, Marco Miceli, Davide Maria Donati, Stefano Fanti, Cristina Nanni, Stefania Benini, Alessandra Longhi, Anna Paioli, Andrea Marrari, Rossella Hakim, Alberto Righi, Toni Ibrahim

**Affiliations:** ^1^ Osteoncology, Soft Tissue and Bone Sarcomas, Innovative Therapy Unit, IRCCS Istituto Ortopedico Rizzoli, Bologna, Italy; ^2^ Department of Pathology, IRCCS Istituto Ortopedico Rizzoli, Bologna, Italy; ^3^ Third Orthopaedic Clinic and Traumatology, IRCCS Istituto Ortopedico Rizzoli, Bologna, Italy; ^4^ Diagnostic and Interventional Radiology, IRCCS Istituto Ortopedico Rizzoli, Bologna, Italy; ^5^ Department of Biomedical and Neuromotor Sciences (DIBINEM), University of Bologna, Bologna, Italy; ^6^ IRCCS Azienda Ospedaliero-Universitaria di Bologna, Policlinico Di Sant’Orsola, Bologna, Italy

**Keywords:** neurotrophic tyrosine receptor kinase, undifferentiated spindle cell sarcoma, bone sarcoma, larotrectinib, entrectinib, NTRK

## Abstract

Background: Neurotrophic tyrosine receptor kinase (NTRK) gene-fusion targeted molecules revolutionized the paradigm of treatment of a limited subgroup of cancers of various histologies. Entrectinib and larotrectinib obtained unprecedented response rates in patients with cancer harboring NTRK rearrangements. This evidence recently led to the agnostic approval of these drugs, and evidence (confirmation) of their activity in a broader disease setting is emerging. Here, we report the case of a patient affected by EML4-NTRK3 rearranged undifferentiated spindle cell bone sarcoma treated with larotrectinib, and we argue (discuss about) the incidence and clinical presentation of NTRK gene-fusion positive bone sarcomas, the potential use of upfront treatment with NTRK inhibitors in neoadjuvant setting, and the role of a multidisciplinary tumor board. Despite the rarity of these rearrangements in patients with primitive bone sarcomas, the therapy with NTRK inhibitors represents a highly effective strategy to be pursued in selected cases even in neoadjuvant settings. The management of these very rare cancers should always be discussed in a multidisciplinary board of reference centers.

## Introduction

Neurotrophic tyrosine receptor kinase 1, 2, and 3 (NTRK1, NTRK2, and NTRK3) genes encode for three tyrosine kinase receptors (TRKA, TRKB, and TRKC, respectively) and act as oncogenic drivers in a well-defined subgroup of cancers (1). Gene fusions involving those genes result in an inappropriately elevated and constitutive tyrosine kinase activity leading to the activation of PI3K/AKT/mTOR, Ras/Raf/MAPK, and PLCγ signaling pathways and eventually to cell proliferation and survival (2, 3). Other types of mutations of NTRK genes, as somatic point mutations, have been reported although even rarer. Although they are not frequent, NRTK rearrangements can occur in a variety of cancers, with a report showing a prevalence of 0.28% on a total of 26,000 cancers with uncommon histology (4, 5).

In soft tissue, infantile fibrosarcoma is characterized by *ETV6:NTRK3* translocations. Moreover, in the last WHO classification of soft tissue and bone tumors, a new category of NTRK-rearranged spindle cell neoplasm (outside infantile fibrosarcoma) has been described as an emerging entity (6). These are soft tissue tumors characterized by a variable spindle cell morphology. Lipofibromatosis-like neural tumors affects children and are histologically characterized by a proliferation of monomorphic spindle cells infiltrating the fat in a reticular pattern. Another subset, affecting a wider age range group, is characterized by a moderate or high cellular spindle cell proliferation with distinctive prominent stromal bands and perivascular rings of keloid-like hyalinized collage. This last subgroup shows a variable histological grading (from low to high grade); it can also show features reminiscent of malignant peripheral nerve sheath tumor (“MPNST-like”) or fibrosarcoma (“FS-like”). All NTRK-rearranged neoplasms are characterized by immunohistochemical positivity for CD34 and S100 and, molecularly, by fusions of *NTRK1*, *NTRK2*, and *NTRK3* genes with variable partners; alternative *RAF1* and *BRAF* fusions have also been described. While lipofibromatosis-like neural tumors are locally aggressive tumors, with no metastatic potential, in the other variants, the prognosis seems to be related to the histological grade, with metastasis to lung and other organs occurring in higher-grade lesions.

Only anecdotal cases of primary bone NRTK-rearranged tumors have been reported (7).

Finally, NTRK rearrangements have been reported in other sarcomas, as 3/21 (14%) of malignant peripheral nerve sheath tumors (MPNST) with NF1 alterations (8), and very rarely (<1%) in other adults sarcoma subtypes (**Table 1**) (5, 9).

**Table 1:** Frequencies of NTRK rearrangements in sarcomas.

Sarcoma subtypes	Percentage	Reference
Overall	0.3%–0.7%	Rosen et al., 2020 (4) Solomon et al., 2020 (9)
Infantile fibrosarcoma	90.6%	Forsythe et al., 2020 (10)
Inflammatory myofibroblastic tumor	17.7%	Solomon et al., 2020 (9)
NF1-related malignant peripheral nerve sheath tumors	14%	Hiemcke-Jiwa et al., 2023 (8)
Osteosarcoma	2.7%	Ameline et al., 2020 (11)
Uterine sarcoma	0.34%–1.1%	Forsythe et al., 2020 (10) Rosen et al., 2020 (4)

MPNST, malignant peripheral nerve sheath tumor.

Larotrectinib, a first-in-class tropomyosin receptor kinase (TRK) inhibitor, achieved a 75% overall response rate (ORR) and a 35-month median progression-free survival (PFS) in a poled analysis of three solid tumor clinical trials (12). Furthermore, in a pediatric soft tissue sarcoma series, the ORR was 94% and median PFS was still not reached (13). In the latter series, one bone sarcoma patient experienced a partial response, and another had stable disease (13, 14). No comprehensive data on bone sarcoma are available.

## Case description

A 33-year-old man with a history of increasing pain at the right foot with functional impairment was admitted to our hospital. The patient reported that the pain arose after an accidental fall. His past medical history was unremarkable. No daily assumption of drugs was referred. The family medical history was not suggestive for increased cancer incidence or cancer predisposition syndrome.

**Figure 1:**
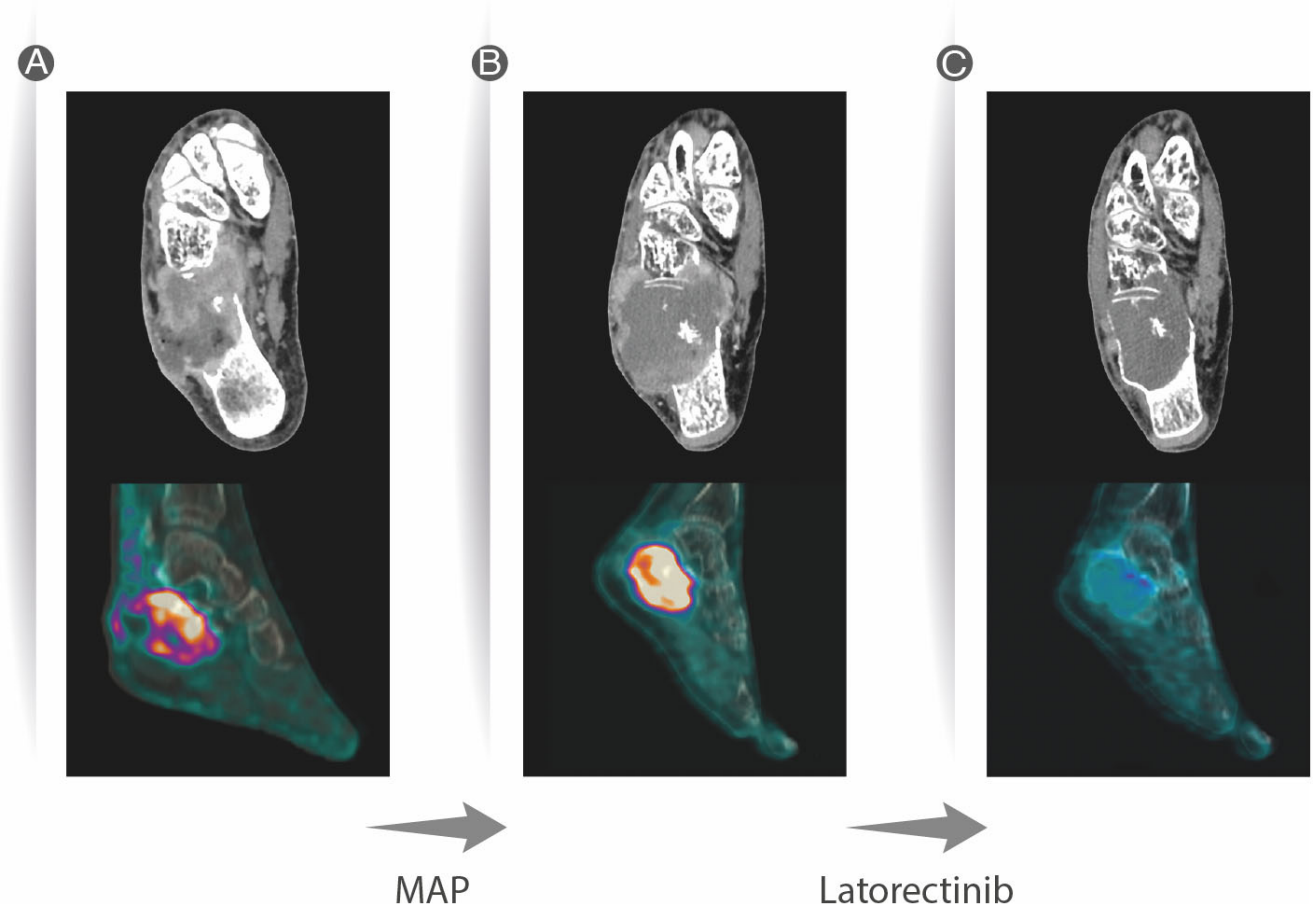
CT scan of the right foot showing an osteolytic lesion of the calcaneus with extensive cortical destruction, with some areas with mild sclerotic reaction and permeative pattern in the anterior aspect. Soft tissue involvement and inhomogeneous contrast enhancement (CE), as well as invasion of the cuboid. Avid FDG uptake was present at PET/CT, with 11.6 Standarized Uptake Value (SUV) **(A)**. Two months after neoadjuvant chemotherapy, an increase in overall size of the lesion was observed at CT scan, associated with increased necrotic component, presenting with more peripheral CE; FDG uptake at PET/CT was still avid **(B)**. Two months after larotrectinib therapy, lesion response showed a significant decrease in size, with reduction of the soft tissue component of the mass, mild and inhomogeneous restoration of cortical margins with increased perilesional sclerotic aspects, and significant decrease in FGD uptake (SUV max 3) **(C)**.

The X-ray showed a large lytic lesion in the calcaneus, without sclerotic rim. The CT scan of the foot showed a large lytic lesion in the calcaneus (**Figure 1A**) and a ^18^FDG-PET/TC scan showed an area of increased uptake (SUV 19.5) in the heel of the right foot (**Figure 1A**). The lesion had a large extra-compartmental component on the soft tissue, near the calcaneus, where it reached the cutaneous plane. The MRI showed an hypointense lesion in T1 and hyperintense T2/T1 fat-sat, respectively.

**Figure 2:**
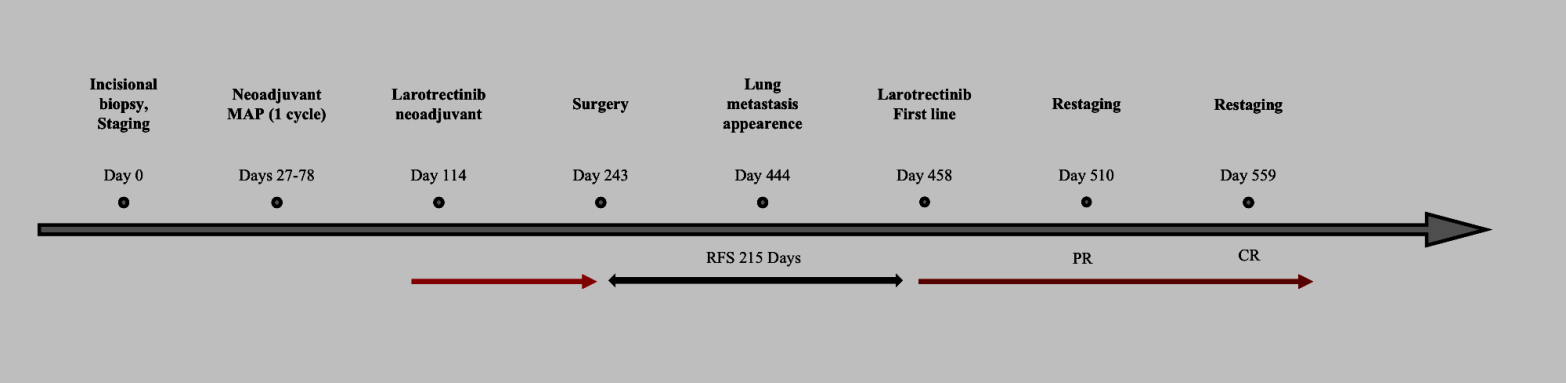
Timeline of events. Red arrow: treatment with larotrectinib ongoing. Black arrow: relapse-free survival (RFS, days). PR, partial response; CR, complete response.

**Figure 3:**
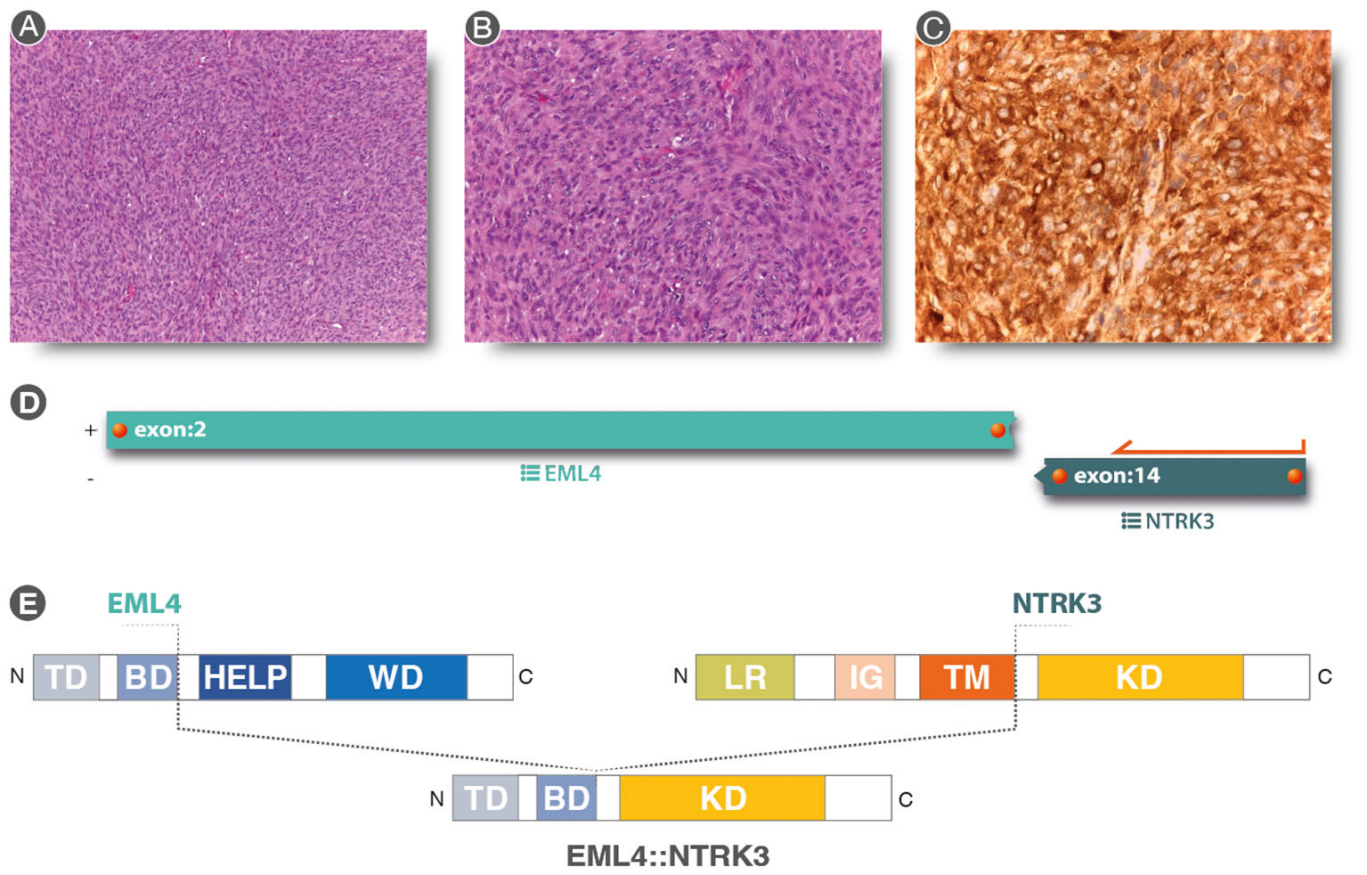
**(A, B)** (Hematoxylin & eosin staining 10× and 20× original magnification, respectively): Spindle cell proliferation with evident nuclear atypia staining and mytotic figures, in a storiform pattern. **(C)** Strong and diffuse cytoplasmic, nuclear membrane, and nuclear staining for pan-TRK immunostaining (20×). **(D)** Analysis of anchored multiplex PCR results of the Archer FusionPlex Sarcoma Panel resulting in a fusion between *EML4* exon2 and *NTRK3* exon 14. **(E)** Graphical representation of *EML4::NTRK3* rearrangement. *EML4::NTRK3* predicted domains showing the contribution of the *EML4* Basic Domain and the complete protein tyrosine kinase domain (KD) of *NTRK3*. Diagram of EML4 domain: TD, trimerization domain; BD, basic domain; HELP, hydrophobic motif in EML proteins; WD, tryptophan-aspartic acid repeats. Diagram of *NTRK3* domains: Leucine-rich (LR) domain, immunoglobulin-like (IG) domain, transmembrane (TM) domain, and kinase domain (KD). The translocation was a result of a fusion between exon 2 of *EML4* and exon 14 of *NTRK3*, resulting in a protein consisting of the Basic Domain of *EML4* and the entire Kinase Domain of *NTRK3*.

The patient underwent an incisional biopsy (**Figure 2**). The morphological aspect and the immunohistochemical and molecular structure were referable to undifferentiated high-grade spindle cell sarcoma (**Figures 3A, B**). The neoplasm showed positivity for Smooth Muscle Actin, MDM2 (focally), and pan-TRK (**Figure 3C**), while Caldesmon, CD31, CK AE1/AE3, Desmin, ERG, S100, SATB2, STAT6, H3F3A (G34V), H3F3A (G34W), H3F3A (G34R), SS18-SSX, SSX, STAT6, and CD34 were negative.

Due to pan-TRK positivity, NGS with FusionPlex Expanded Sarcoma – v1.1 (Archer, Boulder, CO, USA and ThermoFisher Scientific, MA, USA) was performed. *EML4::NTRK3* gene fusion (breakpoint chr2:42472827, chr15:88576276) was detected (**Figures 3D, E**).

Despite the fact that this translocation is in the spectrum of infantile fibrosarcoma, considering the adult age of the patient and the bone location, we considered this tumor as a bone NTRK-rearranged high-grade sarcoma.

No other areas of pathological uptake were reported. The chest CT scan was negative.

After multidisciplinary discussion, the tumor board decision was to start upfront chemotherapy with methotrexate, doxorubicin, and cisplatin (MAP), in analogy to primary conventional high-grade osteosarcoma protocols (15, 16).

Methotrexate 12 g/m^2^ on day 1, followed by cisplatin 120 mg/m^2^ in 48 h continuous infusion (ci) and doxorubicin 75 mg/m^2^ 24 h ci on days 13–16 were administered. The course of treatment was complicated by an episode of transient acute renal failure 24 days after cisplatin treatment.

A new CT scan after 2 months showed enlargement of the lesion (60 × 63 mm vs. 53 × 51 mm) with increase in both the osteolytic component and extra-osseus extension with involvement of neurovascular bundle (**Figure 1B**). Also, clinical worsening of both pain (from 3 to 6 NRS scale) and swelling was reported.

After tumor board discussion, a larotrectinib treatment within a compassionate program was proposed. The baseline ^18^FDG-PET/CT showed the presence of the known calcaneal lesion (SUV 11.6) without any nodal or other sites of distant metastases (**Figure 1B**).

Larotrectinib was administered orally at a dosage of 100 mg twice daily from July to November 2021.

The patient reported progressive decrease of pain and swelling. The ^18^FDG-PET/CT performed after 2 months showed a metabolic response according to PERCIST on the primary lesion (SUV 3.7 vs. 11.6), without any other signs of pathological uptake (**Figure 1C**). The CT scan of the foot showed a partial response according to RECIST 1.1 with sclerotic changes on the cortex and a complete response of the extra compartmental component, which was no more evident (**Figure 1C**). No drug-related toxicity was reported.

After 4 months from the start of the therapy, the patient underwent surgery. A transtibial amputation of the right leg was performed. The CT scan pre-surgery showed a large necrotic component. The histopathological evaluation of the surgical specimen demonstrated an 80% induced tumoral necrosis.

After multidisciplinary discussion, it was concluded that, with no measurable disease, despite a very good clinical and radiological response, no adjuvant larotrectinib should be proposed.

The patient started a follow-up program.

A chest CT scan after 7 months (on June 2022) showed the appearance of two lung metastasis (4.7 and 5.1 mm, respectively). The PET-CT scan ruled out other distant metastases and confirmed the 2 nodules (no uptake due to small size).

Treatment with larotrectinib (100 mg BID) was resumed and a new chest CT scan after 2 months showed a dimensional decrease of both lung nodules.

In the following CT scan, after 4 months, pulmonary lesions were completely disappeared. Currently, the patient is still on treatment with no evidence of disease after 14 months from larotrectinib rechallenge. **Figure 2** shows the patient management timeline.

## Discussion

Spindle cell sarcoma of the bone is a singular entity of mesenchymal tumors usually treated as high-grade osteosarcoma (15, 17, 18). NTRK-rearranged spindle cell neoplasms have been described in the last WHO classification of soft tissue and bone tumors as an emerging entity, occurring in the soft tissues. Only anecdotal cases of primary bone NRTK-rearranged tumors have been reported. Here, we report the case of an unusual NTRK-rearranged high-spindle cell sarcoma, primarily occurring in the bone, responding to larotrectinib, after progression to chemotherapy.

Because of its ultra-rarity, the prevalence of NTRK-positive bone tumor is not known, and differences among reports might also depend on different testing methods. Lam et al. recently reported a 5% positivity rate at immunohistochemical staining in a series of 354 primary bone tumors screened by pan-Trk, but no NTRK fusion was detected at molecular analysis (7). Another report revealed a 2.7% prevalence of NTRK fusion on osteosarcoma patients (11). Siozopoulou et al. identified two NTRK3 fusions out of 70 patients with bone and soft tissue sarcoma and, of note, both the patients were affected by spindle cell tumors (19).

In 2018, larotrectinib was the second molecule to be approved by the FDA on the basis of a molecular target rather than the cancer histotype, after the result of a pivotal phase I–II trial (20). Entrectinib also achieved approval the following year (21).

The use of both of these tissue-agnostic molecules has led to significant results in terms of both disease response and long-term control in the approval trial.

Focusing on sarcoma patients, the response with larotrectinib ranges from 93% in a pediatric study, with a median age of 4.5 years (IQR 1.3–13.3), also including 47% of infantile fibrosarcoma, 41% of soft tissue sarcoma, and 0% bone sarcoma (22), to 79% in a merged analysis of solid tumor also including two sarcoma patients (23).

In our case, larotrectinib was used as neoadjuvant treatment.

While a phase II trial of larotrectinib as a neoadjuvant agent for patients with newly diagnosed infantile fibrosarcoma was launched (24), no data are available on neoadjuvant use in other solid tumors and sarcomas, although a case report on soft tissue NTRK-positive patients was reported (3).

The present case report showed radiological and clinical progression of the disease in a patient with NTRK+ undifferentiated high-grade spindle cell sarcoma treated with chemotherapy, which is a standard for osteosarcoma (15, 16) and used in other spindle cell sarcoma of bone (17). Nonetheless, there was a 40% reduction in maximum SUV (25).

Interestingly, with larotrectinib, there was >60% reduction in maximum SUV (25) and pronounced tumor reduction, both clinical and radiological.

There are several open questions to be addressed on treatment of NTRK+ bone tumors: firstly, the role of targeted therapies in the neoadjuvant setting in addition or replacement to standard chemotherapy; secondly, the duration of neoadjuvant treatment administration, the assessment of tumoral response, and the definition of radiological adequate response to refrain from demolitive surgery; and finally, it is not known if adjuvant therapy should be proposed after surgery, and how long the therapy should be continued after achieving a complete response in the advanced setting.

Considering the present case, we believe that the neoadjuvant NTRK+ inhibitor might be considered upfront. Neoadjuvant treatment duration should be adapted according to metabolic and radiological responses. Long-term results of ongoing trials with larotrectinib in the metastatic setting might support treatment decisions in the future.

Despite the rarity of sarcoma patients harboring NTRK gene fusion, the therapeutical impact of these findings justifies the molecular or immunohistochemical NTRK test in selected cases (5, 14, 26).

This case underlines the relevance of multidisciplinary team discussion, as well as online molecular board support, to guide decision-making in oncology (27, 28).

## Data availability statement

The original contributions presented in the study are included in the article/supplementary material. Further inquiries can be directed to the corresponding author.

## Ethics statement

Ethical approval was not required for the studies involving humans because a written informed consent was obtained from the participant for the publication of data included in this article. The studies were conducted in accordance with the local legislation and institutional requirements. The participants provided their written informed consent to participate in this study. Written informed consent was obtained from the individual(s) for the publication of any potentially identifiable images or data included in this article. An informed consent to publish the information and images of this case report was obtained.

## Author contributions

EP and GF conceived the idea for the paper. MG and AR performed histological diagnosis. SB performed the immunohistochemical and molecular studies. AB, MM, CN, and SF performed image acquisitions. EP and GF drafted the manuscript. All authors read and approved the final version of the manuscript for submission.
